# Identifying Novel Clusters of Patients With Prolonged Mechanical Ventilation Using Trajectories of Rapid Shallow Breathing Index

**DOI:** 10.3389/fmed.2022.880896

**Published:** 2022-07-04

**Authors:** Tsung-Ming Yang, Lin Chen, Chieh-Mo Lin, Hui-Ling Lin, Tien-Pei Fang, Huiqing Ge, Huabo Cai, Yucai Hong, Zhongheng Zhang

**Affiliations:** ^1^Division of Pulmonary and Critical Care Medicine, Chiayi Chang Gung Memorial Hospital, Chiayi, Taiwan; ^2^School of Traditional Chinese Medicine, Chang Gung University, Taoyuan, Taiwan; ^3^Department of Respiratory Care, Chang Gung University of Science and Technology, Chiayi, Taiwan; ^4^Department of Critical Care Medicine, Affiliated Jinhua Hospital, Zhejiang University School of Medicine, Jinhua, China; ^5^Key Laboratory of Emergency and Trauma, Ministry of Education, College of Emergency and Trauma, Hainan Medical University, Haikou, China; ^6^College of Medicine, Graduate Institute of Clinical Medical Sciences, Chang Gung University, Taoyuan, Taiwan; ^7^Department of Nursing, Chang Gung University of Science and Technology, Chiayi, Taiwan; ^8^Department of Respiratory Therapy, Chang Gung University, Taoyuan, Taiwan; ^9^Department of Respiratory Therapy, Chiayi Chang Gung Memorial Hospital, Chiayi, Taiwan; ^10^Department of Respiratory Care, Sir Run Run Shaw Hospital, Zhejiang University School of Medicine, Hangzhou, China; ^11^Key Laboratory of Precision Medicine in Diagnosis and Monitoring Research of Zhejiang Province, Department of Emergency Medicine, Sir Run Run Shaw Hospital, Zhejiang University School of Medicine, Hangzhou, China

**Keywords:** prolonged mechanical ventilation, rapid shallow breathing index, gradient boosting machine, mortality, ICU

## Abstract

**Objective:**

Patients with prolonged mechanical ventilation (PMV) are comprised of a heterogeneous population, creating great challenges for clinical management and study design. The study aimed to identify subclusters of PMV patients based on trajectories of rapid shallow breathing index (RSBI), and to develop a machine learning model to predict the cluster membership based on baseline variables.

**Methods:**

This was a retrospective cohort study conducted in respiratory care center (RCC) at a tertiary academic medical center. The RCC referral criteria were patients with mechanical ventilation for at least 21 days with stable hemodynamic and oxygenation status. Patients admitted to the RCC from April 2009 to December 2020 were screened. Two-step clustering through linear regression modeling and k-means was employed to find clusters of the trajectories of RSBI. The number of clusters was chosen by statistical metrics and domain expertise. A gradient boosting machine (GBM) was trained, exploiting variables on RCC admission, to predict cluster membership.

**Results:**

A total of 1371 subjects were included in the study. Four clusters were identified: cluster A showed persistently high RSBI; cluster B was characterized by a constant low RSBI over time; Cluster C was characterized by increasing RSBI; and cluster D showed a declining RSBI. Cluster A showed the highest mortality rate (72%), followed by cluster D (63%), C (62%) and B (61%; p = 0.005 for comparison between 4 clusters). GBM was able to predict cluster membership with an accuracy of > 0.95 in ten-fold cross validation. Highly ranked variables for the prediction of clusters included thyroid-stimulating hormone (TSH), cortisol, platelet, free thyroxine (T4) and serum magnesium.

**Conclusions:**

Patients with PMV are composed of a heterogeneous population that can be classified into four clusters by using trajectories of RSBI. These clusters can be easily predicted with baseline clinical variables.

## Background

Prolonged mechanical ventilation (PMV) after critical illness has long been noticed as an emerging public health challenge. It is reported that patients with PMV have a 1-year mortality rate of 50–70% (1). This group of patients is typically characterized by old age, high comorbidity burden, high frailty score and increased likelihood of in-hospital complications (2). Great efforts have been made to improve the clinical outcomes of these patients. For example, many hospitals established specialized ventilator weaning unit such as respiratory care center (RCC) to manage these patients (3). In the literature, there have been many studies reporting the epidemiological characteristics of PMV patients, including risk factors for PMV, prediction of weaning probability, short and long-term mortality (4–6). The results are inconsistent across studies due to the heterogeneity of the PMV patients.

While PMV is well described in the literature, it has been noted that PMV patients are heterogeneous, comprising subclusters with distinct clinical characteristics and clinical outcomes. The heterogeneity creates great challenges for the clinical management and study designs. To the best of our knowledge, there has been no study to address the heterogeneity of PMV patients in the literature. Since MV liberation is the primary aim in the management of these patients, many studies have developed models and/or scores for the prediction of ventilator weaning (7–10). Rapid shallow breathing index (RSBI), defined as the ratio of respiratory frequency to tidal volume, is a canonical index to predict weaning success (11, 12). People on a ventilator who cannot tolerate independent breathing tend to breathe rapidly and shallowly and will therefore have a high RSBI. It is reasonable to characterized patients into subclusters based on longitudinal changes of RSBI. The present study aimed to explore the latent subclusters of PMV patients based on the trajectories of RSBI. A machine learning (ML) model based on variables collected upon RCC arrival was trained to predict cluster membership of PMV patients. Important variables associated with cluster assignment were explored in the ML model. We hypothesized that PMV patients could be well separated into several subtypes. The subtypes would have prognostic value for weaning and mortality outcomes. More importantly, these subtypes can be predicted early by using machine learning method trained on routinely collected variables.

## Methods

### Source of Data

This is a retrospective study conducted in the RCC of the Chang Gung Memorial Hospital from April 2009 to December 2020. All patients admitted to the RCC was screened for potential eligibility. The study was approved by the institutional review board (IRB) of the Chang Gung Memorial Hospital (Approval number: 202101862B0). The written informed consent was waived by the IRB because the study did not involve any interventions. Data were deidentified and stored in an encrypted computer. One patient with positive for HIV was excluded for confidential issues. The study was conducted according to the Helsinki declaration and was reported in accordance to the transparent reporting of a multivariable prediction model for individual prognosis or diagnosis (TRIPOD) checklist (13).

### Participants

All patients admitted to the RCC was screened for potential eligibility. The indications for RCC admission must fulfill all the following criteria: (1) patients with mechanical ventilation for at least 21 days; (2) stable hemodynamic status (mean blood pressure > 70 mmHg with normal serum lactate) without vasopressors to maintain blood pressure; (3) stable oxygenation status with FiO2 < 40% and positive end expiratory pressure (PEEP) < 10 cm H_2_O. Patients met one of the following criteria were excluded: (1) duplicated admissions to the RCC of the same patient; (2) patients who declined weaning attempts; (3) withdrawal of life support; (4) Transfer to other facility before weaning attempt started and (5) no spontaneous breathing.

### Patient Characteristics

Demographics, clinical and laboratory variables on RCC entry were extracted from the medical records. Demographic and clinical variables included age, sex, etiology of mechanical ventilation, hospital days upon RCC arrival, ventilation days upon RCC arrival, use of non-invasive ventilation (NIV) upon RCC arrival, Glasgow coma scale (GCS) upon RCC arrival, and comorbidities. Laboratory variables included blood gas, white blood cell count (WBC), hemoglobin (Hb), hematocrit (Hct), mean corpuscular volume (MCV), red cell distribution width (RDW), platelet, segment, lymphocyte, monocyte, eosinophil, basophil, neutrophil to lymphocyte ratio (NLR), blood urea nitrogen (BUN), creatinine (Cr), ionized calcium (Ca), phosphorus (P), magnesium (Mg), albumin, cortisol (AM), cortisol (PM), thyroid-stimulating hormone (TSH), Free thyroxine (T4), pH, blood gas, dead-space fraction, and prealbumin were extracted.

Weaning indices were measured upon RCC arrival and then once a week as part of the routine practices to assess the patient's readiness for weaning, unless the patient was in respiratory distress requiring FiO_2_ of 50% or higher, or in unstable hemodynamic status requiring vasopressor. Before measurement, the patient was disconnected from the mechanical ventilator. A handheld haloscale respirometer (Ferraris Medical, London, UK) was attached to the endotracheal tube to measure the minute ventilation (L/min). The average tidal volume (ml) was obtained by dividing the minute ventilation by the respiratory rate. Rapid shallow breaths index (RSBI) was calculated by dividing the respiratory rate (breaths/min) by average tidal volume in liter. Maximal negative inspiratory pressure (Pimax) was measured by inspiratory force meter (Boehringer Laboratories, Norristown, PA) when the patient was instructed to inhale forcefully and maximally. Finally, we obtained ventilatory parameters including tidal volume, respiratory rate, minute ventilation, maximal negative inspiratory pressure, and RSBI (14).

### Outcome Measurements

The following clinical outcomes were recorded for the study: long-term mortality outcome followed until December 2021, successful weaning from mechanical ventilation on RCC discharge, post-weaning respiratory failure after RCC discharge, days of duration from RCC discharge to respiratory failure, post-weaning respiratory failure before hospital discharge, days of duration from RCC discharge to respiratory failure in hospital, non-invasive mechanical ventilation (NIV) for post-weaning respiratory failure, invasive mechanical ventilation (IMV) for post-weaning respiratory failure, hospital length of stay, weaning and mortality outcome on hospital discharge, and long-term outcome at most recent follow up.

### Two-Step Clustering Through Linear Regression Modeling and K-Means

Two-step clustering through linear regression modeling and k-means was employed to identified clusters of the RSBI trajectories. Each trajectory was represented by the coefficients of an individually fitted linear regression model. The trajectories are then clustered based on the coefficients using k-means clustering (15, 16). The best number of clusters was determined by multiple metrics including log likelihood value, Bayesian information criterion (BIC), and Akaike's information criterion (AIC). We also considered to merge the cluster with fewer than 20 subjects. The trajectories of weaning indices were visualized for each latent cluster.

### Statistical Analysis

Baseline characteristics and laboratory variables were compared across the identified latent clusters. Categorical variables were reported as number (percentage) and were compared across latent clusters with χ^2^ test. Numeric variables were firstly tested for normality distribution and then compared across latent clusters using analysis of variance or Kruskal-Wallis rank sum test as appropriate (17). A *P* < 0.05 was considered as statistical significance.

### Model Development and Cross Validation

To predict RSBI trajectory clusters on RCC admission, we trained a GBM to predict cluster membership. Since the response was multiclass variable, cross entropy was employed as the loss function. The metric accuracy was used to evaluate the model performance in ten-fold cross validation procedure. GBMs build an ensemble of shallow and weak successive trees with each tree learning and improving on the previous (18, 19). The advantage of GBM includes its flexibility in allowing optimization on different loss functions and providing several hyperparameter tuning options that make the function fit very flexible. No data pre-processing is required that GBM often works great with categorical and numerical values as is. The hyperparameters in our GBM include the number of trees (from 1 to 15 at step 1), learning rate (0.1), and the interaction depth (depth of trees: 10, 15, 20, and 25). The minimum number of observations in terminal nodes was set to 30. A grid search strategy was employed to tune the hyperparameters. The accuracy was used in the 10-fold cross validation process for the hyperparameter tuning.

To understand the potential association between risk factors and latent clusters, we reported model specific variable importance for the GBM model. Variable importance is determined by calculating the relative influence of each variable: whether that variable was selected to split on during the tree building process, and how much the squared error (over all trees) improved (decreased) as a result. A greater value of variable importance indicates its higher association with latent clusters. Model interpretation was also performed by using local interpretable model-agnostic explanations (LIME) and iBreakdown algorithms (20). The intuition behind LIME is to learn the behavior of the underlying model (model-agnostic) by perturbing the predictors to see how the predictions change (21, 22). However, the explanation in LIME is additive while some complex relationships between predictors and clusters are non-additive. To address this limitation of LIME, we employed iBreakdown algorithm to detect interactions for instance-level explanations (23). All statistical analyses were performed with R (version 4.1.1).

## Results

### Participants

**Figure 1:**
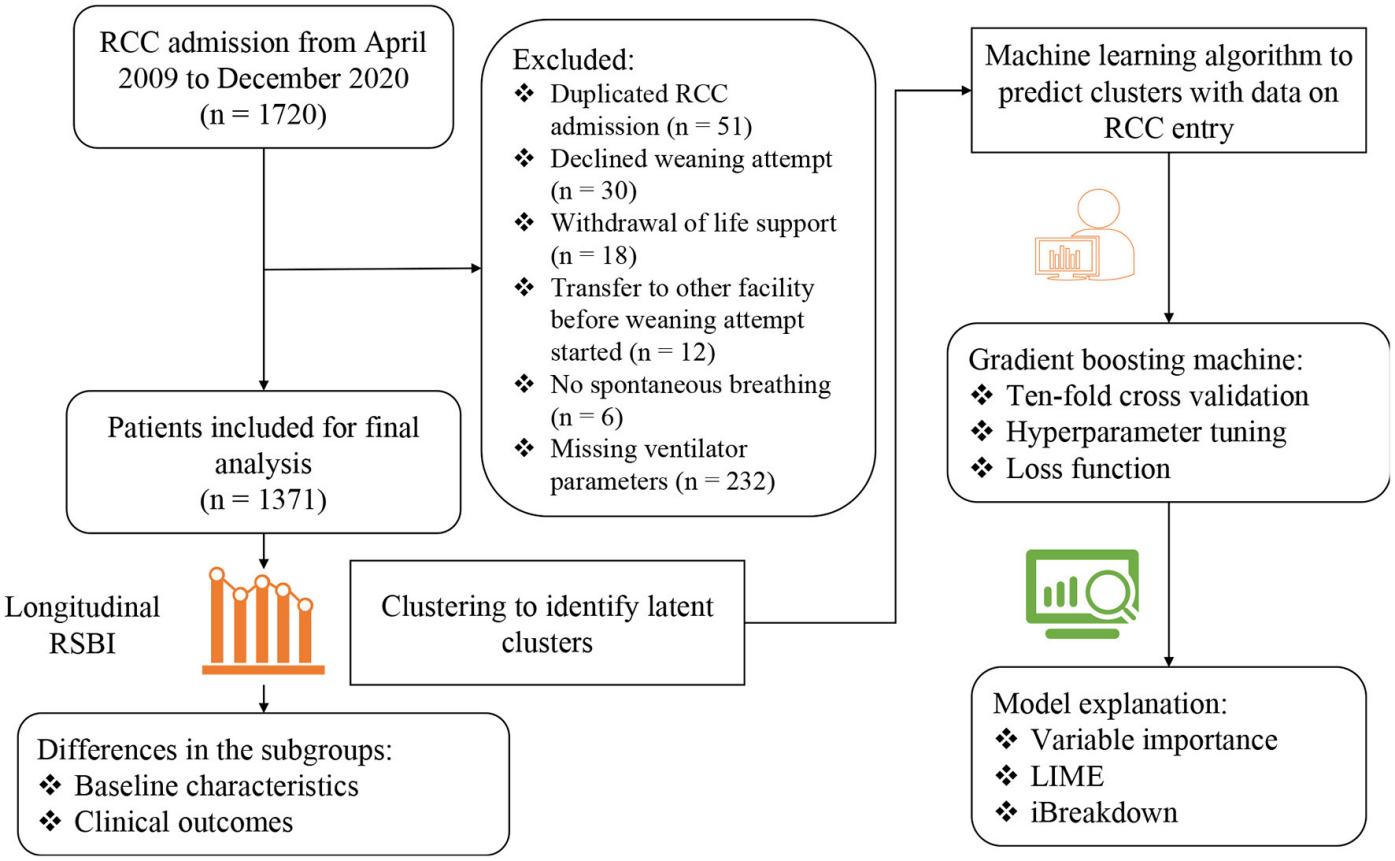
Flowchart of patient enrollment and schematic illustration of the analysis workflow. RSBI, Rapid shallow breathing index; RCC, respiratory care center; LIME, local interpretable model-agnostic explanations.

A total number of 1,720 RCC admissions were screened from April 2009 to December 2020. 349 admissions were excluded due to reasons such as duplicated RCC admission, decline weaning attempt, transfer to other facility before weaning attempt started, no spontaneous breathing, withdrawal of life support and missing data on ventilator parameters (Figure 1). A number of 1,371 RCC admissions were included for the analysis. The median age of the study population was 76 (65–83) years. The median Charlson comorbidity index was 4 (3–7) and the most commonly reason for MV was acute lung injury (37%). The median follow-up days after RCC arrival was 105 (42–512) days (Table 1).

**Table 1:** Baseline characteristics in the total population and across clusters.

**Variables**	**Total (*n* = 1,371)**	**A (*n* = 349)**	**B (*n* = 461)**	**C (*n* = 323)**	**D (*n* = 238)**	***p***
Gender, Male (%)	799 (58)	178 (51)	289 (63)	194 (60)	138 (58)	0.008
Age (years), Median (Q1,Q3)	75.99 (64.89, 82.55)	76.84 (66.39, 82.61)	75.1 (65.08, 82.83)	75.71 (62.3, 82.57)	76.36 (64.41, 82.04)	0.403
APACHE II upon RCC arrival, Median (Q1,Q3)	23 (20, 27)	24 (20, 28)	23 (20, 28)	23 (19.5, 27)	23 (19, 26)	0.021
Tracheostomy, n (%)	371 (27)	94 (27)	127 (28)	92 (28)	58 (24)	0.738
Pre-Albumin (mg/dl, RCC Day 1), Median (Q1,Q3)	16.3 (11.4, 21.4)	16.21 (11.12, 20.65)	16.9 (11.9, 22)	16.2 (11.4, 22.15)	15 (11.52, 21.78)	0.292
Charlson comorbidity index, Median (Q1,Q3)	4 (3, 7)	5 (3, 7)	4 (3, 7)	4 (3, 7)	4 (3, 6)	0.251
GCS upon RCC arrival, Median (Q1,Q3)	9 (7, 11)	9 (7, 11)	9 (7, 11)	10 (8, 11)	10 (9, 11)	0.085
**Etiology of mechanical ventilation, n (%)**						0.107
Acute lung injury	505 (37)	120 (34)	161 (35)	140 (43)	84 (35)	
Neurologic disease	331 (24)	69 (20)	120 (26)	76 (24)	66 (28)	
Miscellaneous	210 (15)	60 (17)	69 (15)	47 (15)	34 (14)	
Cardiac disease	156 (11)	51 (15)	56 (12)	26 (8)	23 (10)	
Post-thoracic or abdominal surgery	100 (7)	27 (8)	30 (7)	22 (7)	21 (9)	
Chronic lung injury	69 (5)	22 (6)	25 (5)	12 (4)	10 (4)	
Equivalent hydrocortisone steroid dose (mg), Median (Q1,Q3)	60 (40, 100)	60 (40, 80)	60 (40, 100)	80 (40, 100)	60 (40, 100)	0.572
Hospital days upon RCC arrival, Median (Q1,Q3)	24 (21, 33)	24 (21, 34)	24 (21, 33)	25 (21, 34)	23 (20, 31)	0.162
Ventilation days upon RCC arrival, Median (Q1,Q3)	21 (20, 25)	21 (20, 25)	21 (20, 26)	22 (20, 25)	21 (20, 24)	0.191
Ventialtor days upon extubation, Median (Q1,Q3)	38 (32, 47)	38 (32, 49)	39 (34, 47)	39 (32, 49)	35 (31, 42)	<0.001
Post-weaning respiratory failure after RCC discharge, n (%)	456 (33)	105 (30)	164 (36)	97 (30)	90 (38)	<0.001
Follow up days after RCC arrival, Median (Q1,Q3)	105 (42, 512)	119 (45, 513)	111 (44, 524)	96 (40, 428)	84 (36, 612.5)	0.542
**Last follow up condition, n (%)**						0.005
Dead	885 (65)	250 (72)	283 (61)	201 (62)	151 (63)	
No ventilator	451 (33)	90 (26)	168 (36)	108 (33)	85 (36)	
On ventilator	35 (3)	9 (3)	10 (2)	14 (4)	2 (1)	
In-hospital mortality, n (%)	363 (26)	86 (25)	125 (27)	84 (26)	68 (29)	0.735
Hospital length of stay, Median (Q1,Q3)	65 (53, 82)	65 (54, 83)	65 (55, 81)	65 (56, 86)	61 (49, 76.75)	0.019
Weaning from MV in hospital or RCC, n (%)	654 (48)	141 (40)	239 (52)	151 (47)	123 (52)	0.007
IMV for post-weaning respiratory failure, n (%)	283 (21)	67 (19)	99 (21)	57 (18)	60 (25)	0.231

*IMV, invasive mechanical ventilation; Q1, the first quartile; Q3, the third quartile; RCC, respiratory care center; GCS, Glasgow coma scale; APACHE II, The Acute Physiology and Chronic Health Evaluation II*.

### Clusters of RSBI Trajectory

**Figure 2:**
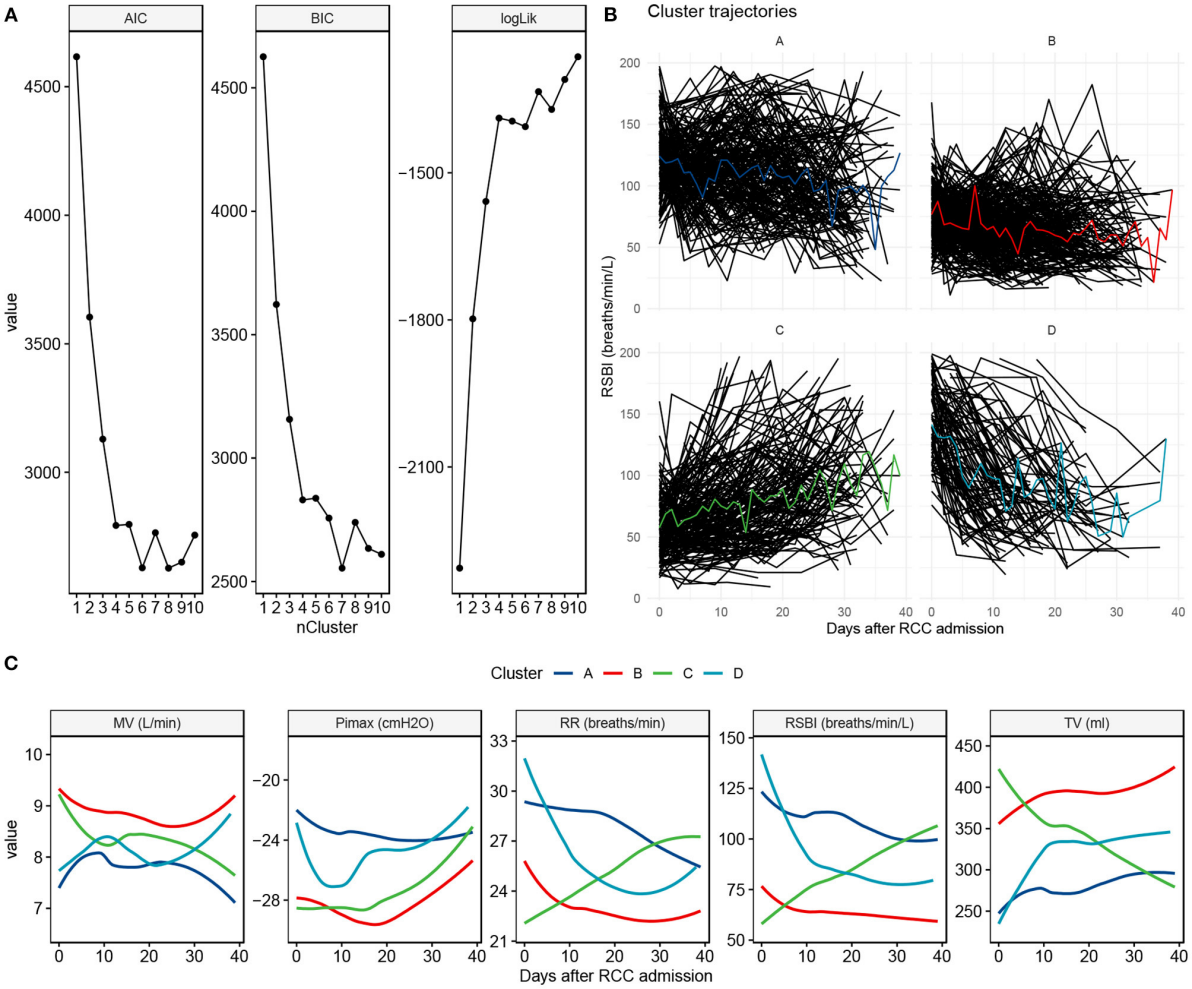
Clustering to identify clusters of patients with prolonged mechanical ventilation. **(A)** The best number of clusters was chosen by using statistical metrics. Greater values of log likelihood indicate better model fit, whereas lower values of BIC and AIC indicate better model fit. **(B)** Trajectory characteristics of each cluster. Individual trajectories are represented by black lines and the cluster trajectory is colored. The cluster label and percentage are shown on the top of each panel. **(C)** Trajectory and 90% confidence interval for each of the ventilator parameters, stratified by the cluster membership. BIC, Bayesian information criterion; AIC, Akaike's information criterion; MV, minute ventilation; Pimax, maximum inspiratory pressure; RR, respiratory rate; RSBI, Rapid shallow breathing index; TV, tidal volume.

The 4-cluster model was considered as the best model it showed low BIC and AIC values, and high Log likelihood value (Figure 2A). Cluster B accounted for the largest proportion of patients and showed a constantly low RSBI during RCC stay. Cluster C was characterized by increasing RSBI (Figures 2B,C).

The clinical characteristics were compared across the clusters. Cluster B showed the highest proportion of male, while cluster D showed the lowest proportion of male patients (63% vs. 51%; p = 0.008). The APACHE II upon RCC arrival was the highest in cluster A and was the lowest in cluster D [median [Q1, Q3]: 24 (20, 28) vs. 23 (19, 26); *p* = 0.021, Table 1]. Interestingly, patients in cluster C showed lower plasma magnesium on RCC entry than that in cluster A (1.87 (1.63, 2.17) vs. 1.99 (1.72, 2.27) mg/dl; *p* = 0.007). The serum cortisone level on RCC entry was also associated with subsequent trajectory clusters (Table 2).

**Table 2:** Laboratory findings on RCC entry.

**Variables**	**Total (*n* = 1,371)**	**A (*n* = 349)**	**B (*n* = 461)**	**C (*n* = 323)**	**D (*n* = 238)**	***p***
WBC (× 10^9^/*L*), median (Q1,Q3)	9.3 (7.05, 12.4)	9.2 (7.1, 12.1)	9.4 (7, 12.5)	9.5 (7.1, 12.55)	9.35 (7.12, 12.2)	0.943
Hb (mg/dl), median (Q1,Q3)	9.7 (8.9, 10.5)	9.6 (8.9, 10.3)	9.7 (9, 10.6)	9.7 (8.9, 10.45)	9.75 (8.9, 10.7)	0.396
Hct, median (Q1,Q3)	0.3 (0.28, 0.32)	0.3 (0.28, 0.32)	0.3 (0.28, 0.33)	0.3 (0.27, 0.32)	0.3 (0.28, 0.33)	0.349
MCV, median (Q1,Q3)	90.6 (87, 94.3)	90.9 (87.1, 95)	90.5 (87.1, 93.9)	90.5 (86.65, 93.7)	90.5 (87.23, 94.27)	0.339
RDW, median (Q1,Q3)	0.16 (0.15, 0.18)	0.16 (0.15, 0.18)	0.16 (0.15, 0.18)	0.16 (0.15, 0.18)	0.16 (0.15, 0.17)	0.024
Platelet (× 10^9^/*L*), median (Q1,Q3)	218 (147, 307.5)	210 (138, 299)	224 (154, 314)	213 (142, 295)	223.5 (160.5, 316.5)	0.057
Segment (× 10^9^/*L*), median (Q1,Q3)	0.79 (0.72, 0.86)	0.79 (0.71, 0.86)	0.79 (0.72, 0.85)	0.8 (0.73, 0.86)	0.79 (0.73, 0.86)	0.9
Lymohocyte (× 10^9^/*L*), median (Q1,Q3)	0.1 (0.06, 0.16)	0.11 (0.06, 0.15)	0.1 (0.06, 0.16)	0.1 (0.07, 0.15)	0.11 (0.06, 0.16)	0.946
Monocyte (× 10^9^/*L*), median (Q1,Q3)	0.06 (0.04, 0.08)	0.06 (0.04, 0.08)	0.06 (0.04, 0.08)	0.06 (0.04, 0.08)	0.06 (0.04, 0.08)	0.893
Eosinophil (× 10^9^/*L*), median (Q1,Q3)	0.01 (0, 0.03)	0.01 (0, 0.03)	0.01 (0, 0.03)	0.01 (0, 0.03)	0.01 (0, 0.03)	0.753
Basophil (× 10^9^/*L*), median (Q1,Q3)	0 (0, 0)	0 (0, 0)	0 (0, 0)	0 (0, 0)	0 (0, 0)	0.571
NLR, median (Q1,Q3)	7.5 (4.66, 13)	7.41 (4.79, 13.23)	7.55 (4.62, 12.43)	7.8 (4.66, 12.91)	7.45 (4.52, 13.42)	0.936
BUN (mg/dl), median (Q1,Q3)	27.8 (16.3, 54)	32.1 (17.5, 59.4)	27.7 (15.9, 54.9)	26.2 (16.55, 50.55)	25.3 (15.93, 49.3)	0.174
Cr (mg/dl), median (Q1,Q3)	0.75 (0.48, 1.71)	0.78 (0.47, 1.69)	0.75 (0.49, 1.93)	0.73 (0.46, 1.79)	0.74 (0.47, 1.46)	0.652
Ca (mg/dl), median (Q1,Q3)	8.2 (7.9, 8.7)	8.3 (7.9, 8.8)	8.3 (7.9, 8.7)	8.2 (7.8, 8.6)	8.2 (7.9, 8.6)	0.23
P (mg/dl), median (Q1,Q3)	3.5 (2.9, 4.2)	3.6 (2.9, 4.2)	3.5 (2.9, 4.2)	3.5 (2.9, 4.4)	3.4 (2.8, 4.1)	0.59
Mg (mg/dl), median (Q1,Q3)	1.91 (1.68, 2.2)	1.99 (1.72, 2.27)	1.88 (1.67, 2.18)	1.87 (1.63, 2.17)	1.92 (1.72, 2.17)	0.007
Albumin (mg/dl), median (Q1,Q3)	2.5 (2, 2.9)	2.4 (2, 2.8)	2.5 (2.1, 2.9)	2.5 (2.02, 2.9)	2.5 (2, 2.8)	0.089
Cortisol (mcg/dl, AM), median (Q1,Q3)	14.32 (10.39, 18.15)	14.51 (10.62, 19.12)	14.97 (10.89, 18.2)	13.91 (10.66, 17.91)	13.04 (9.33, 16.86)	0.013
Cortisol (mcg/dl, PM), median (Q1,Q3)	15.07 (10.6, 20.03)	15.45 (10.52, 20.52)	14.78 (10.72, 20.01)	15.46 (11.31, 20.03)	14.5 (10.19, 18.98)	0.313
TSH (mIU/L), median (Q1,Q3)	2.19 (1.18, 4.24)	2.51 (1.24, 4.46)	2.05 (1.09, 4.32)	2.11 (1.17, 4.18)	2.13 (1.19, 3.9)	0.26
Free T4 (Free T4), median (Q1,Q3)	0.97 (0.8, 1.16)	0.95 (0.79, 1.13)	0.98 (0.8, 1.16)	0.98 (0.82, 1.17)	0.99 (0.8, 1.14)	0.737
pH (Upon RCC arrival), median (Q1,Q3)	7.49 (7.46, 7.52)	7.49 (7.45, 7.51)	7.49 (7.46, 7.52)	7.49 (7.46, 7.52)	7.49 (7.46, 7.52)	0.178
PaCO2 (mmHg, Upon RCC arrival), median (Q1,Q3)	38 (32.92, 43.18)	38.45 (33.7, 44.42)	37.5 (32.4, 42.6)	38 (32.75, 42.8)	37.8 (33.12, 43.1)	0.072
PaO2 (mmHg, Upon RCC arrival), median (Q1,Q3)	101.8 (84.53, 121.92)	101 (85.6, 120.12)	101.3 (83.1, 123.5)	103.8 (86.45, 124.45)	102 (87.82, 119.65)	0.607
HCO3 (mmol/L, Upon RCC arrival), median (Q1,Q3)	29.3 (25.4, 32.9)	29.7 (25.5, 33.6)	29 (25.4, 32.6)	29.1 (25.35, 32.65)	29.65 (25.92, 33.2)	0.263
SaO2 (Upon RCC arrival), median (Q1,Q3)	0.98 (0.97, 0.99)	0.98 (0.97, 0.99)	0.98 (0.97, 0.99)	0.98 (0.97, 0.99)	0.98 (0.97, 0.99)	0.64
FiO2 (Upon RCC arrival), Median (Q1,Q3)	0.35 (0.3, 0.35)	0.35 (0.35, 0.35)	0.35 (0.3, 0.35)	0.35 (0.3, 0.35)	0.35 (0.35, 0.35)	0.268
End-tidal CO2 (mmHg, Upon RCC arrival), median (Q1,Q3)	34 (30, 38)	33 (31, 41)	34 (31, 38)	33.5 (29, 37)	34.5 (30.75, 38)	0.578
Dead space fraction (Upon RCC arrival), mean ± SD	0.08 ± 0.18	0.1 ± 0.17	0.08 ± 0.19	0.08 ± 0.17	0.08 ± 0.18	0.858
Pre-Alb (mg/dl, RCC Day 1), median (Q1,Q3)	16.3 (11.4, 21.4)	16.21 (11.12, 20.65)	16.9 (11.9, 22)	16.2 (11.4, 22.15)	15 (11.52, 21.78)	0.292
Pre-Alb (mg/dl, RCC Day 14), median (Q1,Q3)	17.85 (13.1, 23.7)	17.9 (13.5, 23.85)	17.75 (13.03, 23.62)	16.65 (12.23, 22.2)	20 (15.5, 25.2)	0.022

*WBC, white blood cell count; Hb, hemoglobin; Hct, hematocrit; RCC, respiratory care center; Q1, the first quartile; Q3, the third quartile; BUN, blood urea nitrogen; Cr, creatinine; RDW, red distribution width; MCV, mean corpuscular volume; NLR, neutrophil to lymphocyte ratio; P, phosphorus; Mg, magnesium; TSH, thyroid-stimulating hormone; T4, thyroxine; PaCO2, arterial partial pressure of carbon dioxide; PaO2, arterial partial pressure of oxygen; HCO3, Bicarbonate; SaO2, arterial oxygen saturation; FiO2, inspired oxygen fraction; SD, standard deviation*.

There were significant differences in clinical outcomes between the four clusters (Table 1). For the mortality outcome, cluster B showed the lowest mortality rate and cluster A showed the highest mortality (72 vs. 61%; *p* = 0.005). The weaning probability was highest in cluster B and the lowest in cluster A on hospital discharge (52 vs. 40%; *p* = 0.007). However, there was no significant difference on respiratory failure rate across clusters after successful weaning (p = 0.231).

### Predicting Trajectory Clusters on RCC Admission

**Figure 3:**
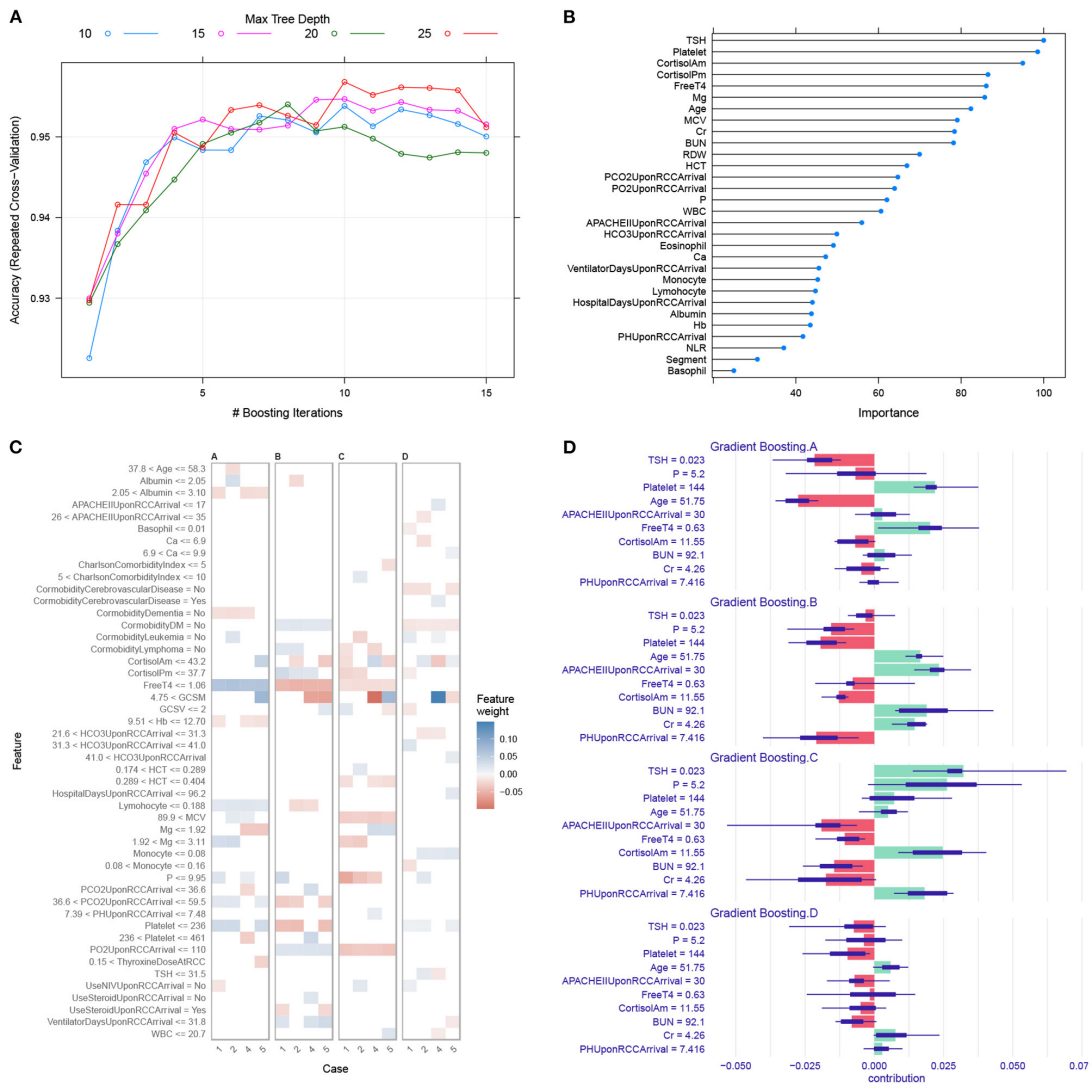
Gradient boosting machine training and interpretation. **(A)** Hyperparameter tuning for the gradient boosting machine model. We used grid search strategy to select hyperparameters with the highest accuracy. **(B)** Variable importance in the GBM model. Higher importance value indicates greater influence of the variable in differentiating the clusters. **(C)** LIME interpretation for four sample subjects. The horizontal axis is labeled by the sample ID. The observed cluster membership for patients 1, 2, 4 and 5 were A, B, C and D respectively. Blue (red) color indicates the variable is supporting for (contradicting against) a given cluster. For example, the subject 4 has magnesium <1.92 supporting for cluster C. **(D)** The iBreakdown explainer for patient #4 showed that there was more support for allocation to cluster C than to other clusters. The feature TSH = 0.023 strongly supports its assignment to cluster C, whereas the APACHE II = 30 on RCC arrival contradicts its assignment to cluster C. The short bar indicates the confidence interval for uncertainty. LIME, local interpretable model-agnostic explanations; HCT, hematocrit; WBC, white blood cell count; BUN, blood urea nitrogen; Cr, creatinine; RDW, red distribution width; MCV, mean corpuscular volume; GCS, Glasgow coma scale; GCSM, motion component of GCS.

The model hyperparameters of the GBM model were chosen by grid search to achieve the highest accuracy (> 0.95; Figure 3A). The top variables that are predictive of trajectory clusters included age, serum cortisol, BUN, platelet, and serum magnesium upon RCC arrival (Figure 3B). Four representative samples (sample ID = 1, 2, 4 and 5) were explored by LIME algorithm, which showed variables supporting or contradicting the assignment to a specific cluster (Figures 3C,D). The result indicated that TSH, cortisol, platelet, free T4 and serum magnesium were important predictors of clusters in many instances.

**Figure 4:**
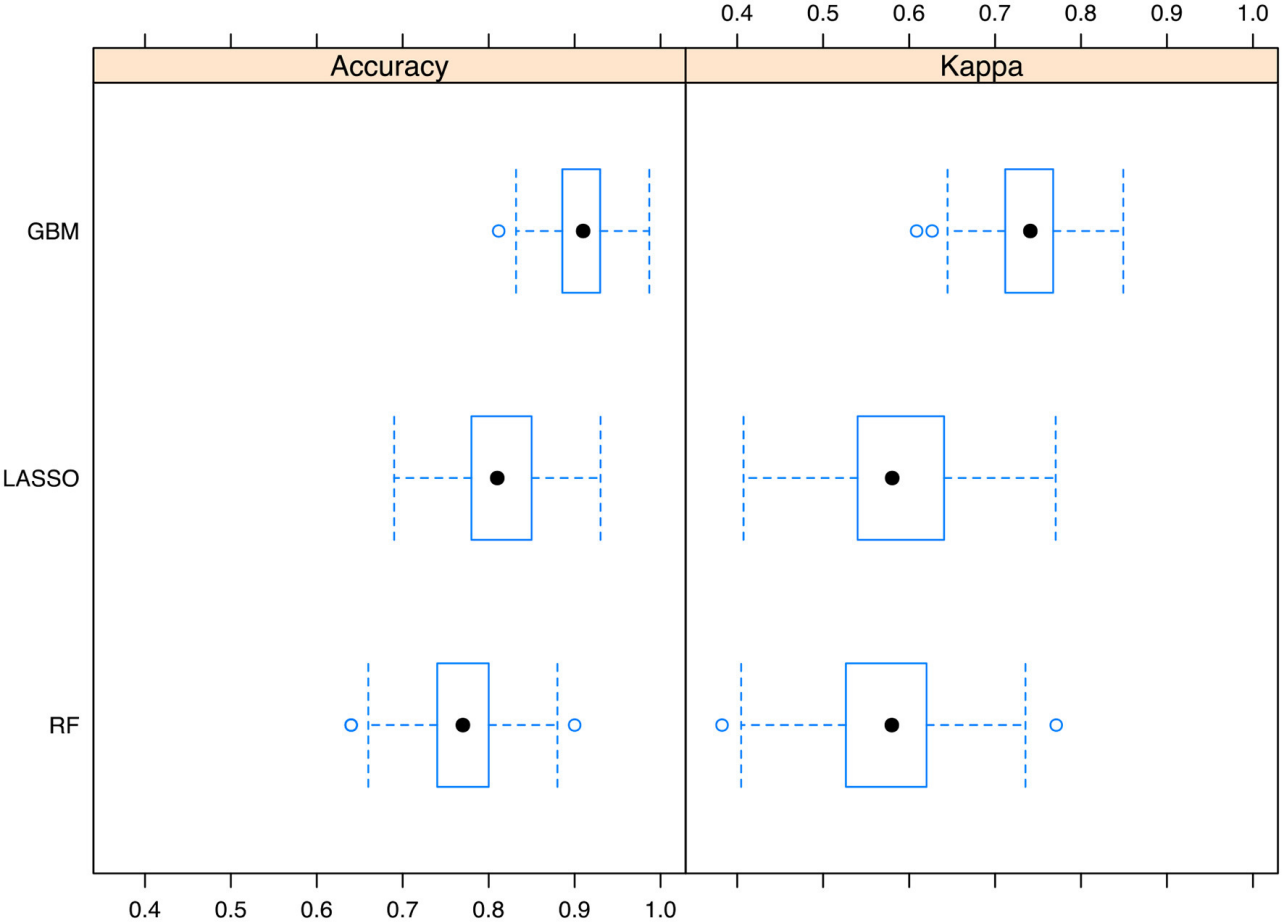
Comparisons of the Gradient boosting machine with other models. The performance metrics of accuracy and kappa was reported. The boxplot shows the median and range of the performance metrics in resampled datasets. GBM, gradient boosting machine; LASSO, Least Absolute Shrinkage and Selection Operator; RF, random forest.

We also trained random forest (RF) and LASSO regression models, against which the GBM model was compared. The results showed that the GBM model outperformed LASSO and RF models with resampling method (Figure 4).

## Discussion

The study for the first time explored the latent trajectories of patients with PMV (IMV duration > 21 days with stable hemodynamic and respiratory conditions) using RSBI. Four clusters were identified for the study population, namely, cluster A, B, C and D. Cluster B was characterized by a constant low RSBI over time; Cluster C was characterized by increasing RSBI; cluster D showed a declining RSBI, and cluster A showed persistently high RSBI. Many variables on RCC entry were associated with cluster membership including TSH, cortisol, platelet, free T4 and serum magnesium. These variables were also confirmed to be top ranked variables in GBM to classify trajectory clusters. It is feasible to predict the trajectories of RSBI upon RCC arrival using machine learning methods. Further external validation of the GBM is mandatory before this model can be used in clinical practice.

The identification of clusters for PMV patients has several implications. First, the heterogeneity of the population is addressed by classifying patients into clinically meaningful subgroups. These subgroups showed distinct clinical characteristics and outcomes, which is helpful for risk stratification and clinical decision making (24). Cluster A showed the lowest survival probability as compared to other clusters. Since it is feasible to predict patient trajectory on RCC admission, such early risk stratification can help resource allocation and family consultation. Second, individualized treatment strategy can be implemented for different subgroups. For example, we observed that low serum magnesium was associated with increased risk of cluster C trajectory with worsening RBSI during RCC treatment. This unfavorable outcome might be addressed by supplementing magnesium for this group of patients. Third, the identification of subtypes of patients can help to design clinical trials. Some interventions may have beneficial effects in a subgroup of patients, and trials investigating such interventions should target this subgroup. Such implementation of trial design has been explored in sepsis, showing that the probability of obtaining statistically significant beneficial/harmful effects vary by the proportion of subtypes (25).

The associations of several variables with cluster membership are supported by the literature. Serum magnesium has long been noticed to be associated with prolonged mechanical ventilation (26, 27). Hypomagnesemia is common in mechanically ventilated patients, and there is strong, consistent observational evidence that hypomagnesemia is significantly associated with increased need for prolonged mechanical ventilation and increased mortality (28). The causality of hypomagnesemia and PMV has not been firmly established in the critical care literature. In a randomized controlled trial involving liver transplantation, Gucyetmez B and colleagues reported that intravenous magnesium sulfate administration was associated with shortened duration of mechanical ventilation (29). However, the association of magnesium and trajectory clusters in RCC has not been explored and this is a novelty in our study. Although our latent clusters were identified by using longitudinal RSBI, changes of other ventilator parameters also have important clinical implications. For example, the cluster A shows constant RR with slightly increasing Pimax (i.e., less negative value indicates less inspiratory efforts) over RCC treatment, indicating less demand of ventilation with recovered critical illness. It is reasonable to deduce that oxygen consumption will decline after resolution of critical illness, which is reflected by reduced minute ventilation. Collectively, these changes in ventilator parameters indicate recovered overall condition and improved lung function.

Several limitations must be acknowledged in the study. First, the study was retrospective in design and there are many missing values in ventilator parameters. We had to exclude these patients due to missingness. It is largely unknown whether this exclusion will compromise the representativeness of our sample for the study population. Second, although we trained a GBM model for the prediction of subsequent trajectory clusters, the model was not validated in external dataset. We used 10-fold cross validation for training the model, but this cannot preclude the possibility of poor performance in other datasets. Third, the causality of baseline characteristic variables and cluster assignment cannot be fully confirmed in the present study design due to potential unmeasured confounding factors. Further randomized controlled trials are mandatory to confirm potential causal associations. P1.0 is another important parameter to predict weaning failure. It was not included in the analysis because this variable was not routinely measured. Finally, RSBI was the primary index used for trajectory clustering, which had its inherent strengths and limitations. RSBI is widely used to predict the weaning success and its measurement is easy at bedside. However, RSBI can be affected by pressure augmentation, PEEP, and a bias flow (30, 31).

## Conclusions

The study identified four clusters of patients requiring PMV based on longitudinal RSBI. These clusters have distinct clinical characteristics and outcomes, which is implicative for the implementation of precise medicine for this study population. It is also feasible to predict cluster assignment with variables collected upon RCC arrival with machine learning algorithms.

## Data Availability Statement

The raw data supporting the conclusions of this article will be made available by the authors, without undue reservation.

## Ethics Statement

The studies involving human participants were reviewed and approved by the Institutional Review Board (IRB) of the Chang Gung Memorial Hospital (Approval number: 202101862B0). Written informed consent for participation was not required for this study in accordance with the national legislation and the institutional requirements.

## Author Contributions

T-MY and ZZ designed the study and drafted the manuscript. H-LL helped interpret the results and write some discussions. ZZ and LC performed statistical analysis and result interpretation. YH and HC prepared the figures and interpret the results. T-PF, C-ML, and HG provided critical review of the manuscript. T-MY, T-PF, and C-ML were responsible for patient enrolment and data entry. ZZ is identified as the guarantor of the paper and taking responsibility for the integrity of the work as a whole, from inception to published article. All authors contributed to the article and approved the submitted version.

## Funding

ZZ had received funding from Yilu Gexin–Fluid Therapy Research Fund Project (YLGX-ZZ-2020005) and the Health Science and Technology Plan of Zhejiang Province (2021KY745). LC had received funding from Zhejiang Provincial Project of Medical and Health Technology (2022PY099), Youth Foundation of Jinhua Municipal Central Hospital (JY2020-2-10), Key Project of Jinhua City (2021-3-037), Key Laboratory of Emergency and Trauma (Hainan Medical University), and Ministry of Education (KLET-202118). H-LL had received funding from Chang Gung Memorial Foundation (BMRPE83).

## Conflict of Interest

The authors declare that the research was conducted in the absence of any commercial or financial relationships that could be construed as a potential conflict of interest.

## Publisher's Note

All claims expressed in this article are solely those of the authors and do not necessarily represent those of their affiliated organizations, or those of the publisher, the editors and the reviewers. Any product that may be evaluated in this article, or claim that may be made by its manufacturer, is not guaranteed or endorsed by the publisher.

## Abbreviations

RCC: respiratory care center
RSBI: Rapid shallow breathing index
IMV: invasive mechanical ventilation
WBC: white blood cell count
RCC: respiratory care center
Q1: the first quartile
Q3: the third quartile
BUN: blood urea nitrogen
Cr: creatinine
RDW: red cell distribution width
MCV: mean corpuscular volume
NLR: neutrophil to lymphocyte ratio
SD: standard deviation
LIME: local interpretable model-agnostic explanations.
